# Assessment for Perioperative Hyperglycemia Prior to Total Joint Replacement in Patients With and Without Diabetes

**DOI:** 10.1001/jamanetworkopen.2019.10589

**Published:** 2019-09-04

**Authors:** Lindsey A. MacFarlane, Yinzhu Jin, Patricia D. Franklin, Joyce Lii, Jeffrey N. Katz, Seoyoung C. Kim

**Affiliations:** 1 Orthopedic and Arthritis Center for Outcomes Research, Department of Orthopedic Surgery, Brigham and Women’s Hospital, Boston, Massachusetts; 2 Division of Rheumatology, Immunology and Allergy, Brigham and Women’s Hospital, Boston, Massachusetts; 3 Harvard Medical School, Boston, Massachusetts; 4 Division of Pharmacoepidemiology and Pharmacoeconomics, Brigham and Women’s Hospital, Boston, Massachusetts; 5 Department of Medical Social Sciences, Northwestern University, Chicago, Illinois

## Abstract

This cohort study examines rates of screening for hyperglycemia before total joint replacement among Medicare enrollees with and without diabetes.

## Introduction

More than 1 000 000 total joint replacements (TJRs) are performed annually in the United States,^1^ most of which are for osteoarthritis.^2^ Diabetes is a frequent comorbidity in patients with osteoarthritis^3^ and suboptimal glucose control preoperatively is associated with poor TJR outcomes.^4,5^ Despite the concern for hyperglycemia in the period before TJR, there is a paucity of data regarding the frequency of preoperative outpatient screening. We aimed to assess how frequently hemoglobin A_1c_ (HbA_1c_) was measured 90 days prior to TJR among Medicare enrollees.

## Methods

We conducted a cohort study using claims data from Medicare Parts A (hospital), B (medical), and D (pharmacy) from January 2010 to December 2014; data were analyzed from May 2018 to July 2019. The index date was the date of first TJR (total hip or knee) during the study period. Patients were aged 65 years or older and were continuously enrolled in Medicare for at least 360 days prior to the index date.

This study was approved by the Brigham and Women’s Health institutional review board, which waived the requirement for obtaining patients’ consent because data were deidentified and patients incurred minimal risk. We followed the Strengthening the Reporting of Observational Studies in Epidemiology (STROBE) reporting guideline.

*International Classification of Diseases, Ninth Revision, Clinical Modification *(*ICD-9-CM*) or *Current Procedural Terminology* codes were used to identify diabetes, complications of diabetes (nephropathy, neuropathy, retinopathy, or foot problems associated with diabetes), TJR, and comorbidities. Using claims from the 270 days prior to the 90-day outcome period, we created 4 mutually exclusive groups: (1) no diabetes (no *ICD-9* code for diabetes or diabetes complications and no claim for insulin or other antidiabetic medications); (2) diabetes and not receiving medication (≥1 *ICD-9* code for diabetes but no claim for insulin or other antidiabetic medications); (3) diabetes and receiving noninsulin medications for diabetes (≥1 *ICD-9* code for diabetes and ≥1 claim for noninsulin antidiabetic medications); and (4) diabetes and receiving insulin (≥1 *ICD-9* code for diabetes and ≥1 claim for insulin).

The primary outcome was the proportion of patients who had an HbA_1c_ level tested in the 90 days prior to TJR. We also assessed the proportion of patients who had a code for serum blood glucose level tested separately or in metabolic panels in the 90 days preceding TJR.

## Results

We had access to 1 046 660 claims for TJR; of these, 465 566 patients met the inclusion criteria (Figure). Among the groups, mean age ranged from 73 to 75 years and 64% to 68% were female (Table). In the 90 days prior to TJR, 4.9% (95% CI, 4.8%-5.0%) of patients without diabetes had HbA_1c_ testing compared with 25.8% (95% CI, 25.4%-26.2%) of those with diabetes not receiving medication, 39.0% (95% CI, 38.6%-39.4%) of those with diabetes receiving noninsulin medications, and 43.4% (95% CI, 42.8%-44.1%) of those with diabetes receiving insulin. Serum glucose testing was performed in 37.2% (95% CI, 37.0%-37.4%) of those without diabetes, 45.7% (95% CI, 45.2%-46.1%) of those with diabetes not receiving medication, 47.7% (95% CI, 47.3%-48.1%) of those with diabetes receiving noninsulin medications, and 50.2% (95% CI, 49.5%-50.9%) of those with diabetes receiving insulin. The proportion of patients with HbA_1c_ or serum glucose level tested was 37.6% in those without diabetes, 48.6% in those with diabetes not receiving medication, 52.9% for those with diabetes receiving noninsulin medication, and 56.8% for those with diabetes receiving insulin.

**Figure.**
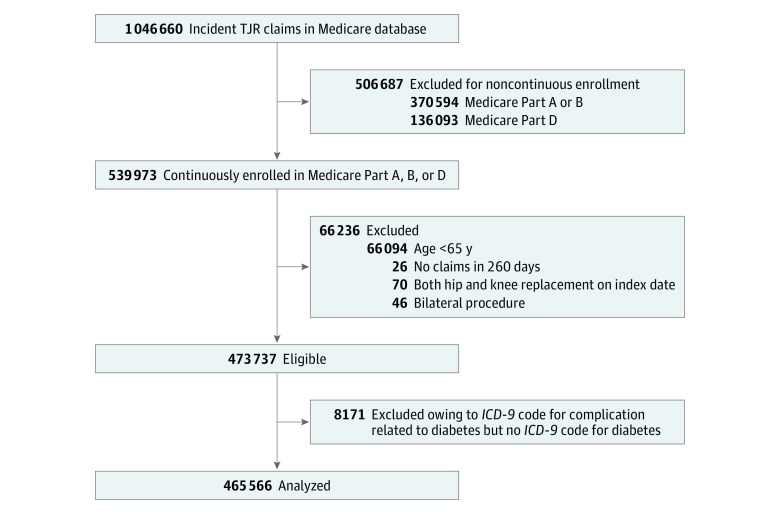
Cohort Selection Flow Diagram The criteria for exclusions were checked consecutively. *ICD-9* indicates *International Classification of Diseases, Ninth Revision*; TJR, total joint replacement.

**Table.** Baseline Characteristics of Study Cohort^a^

Characteristic	No. (%)			
	No Diabetes (n = 335 365)	Diabetes (n = 130 201)		
		Without Medication (n = 49 965)	Noninsulin Medication (n = 59 705)	Insulin (n = 20 531)
Age, mean (SD), y	74 (6)	75 (6)	74 (6)	73 (6)
No. of physician visits, median (IQR)	7 (4-10)	9 (5-13)	8 (5-12)	10 (6-15)
Total No. of unique prescription medications, median (IQR)	7 (4-10)	9 (6-13)	11 (8-14)	14 (11-18)
Female	229 306 (68)	33 031 (66)	37 957 (64)	13 126 (64)
Race/ethnicity				
Black	11 745 (4)	3313 (7)	4445 (8)	2036 (10)
Hispanic	3252 (1)	956 (2)	1482 (3)	516 (3)
White	312 919 (94)	43 942 (89)	51 431 (88)	17 328 (86)
Other	3516 (1)	1024 (2)	1343 (2)	365 (2)
Missing	3933 (1)	730 (1)	1004 (2)	286 (1)
Complications of diabetes				
Nephropathy	NA	1521 (3)	3099 (5)	3000 (15)
Neuropathy	NA	4350 (9)	10 419 (18)	6896 (34)
Retinopathy	NA	1777 (4)	5908 (10)	5024 (25)
Foot problems	NA	1564 (3)	1573 (3)	1233 (6)
Treatment of diabetes				
Biguanide	NA	NA	47 944 (80)	8405 (41)
Sulfonylureas	NA	NA	18 082 (30)	3805 (19)
Thiazolidinediones	NA	NA	7521 (13)	1436 (7)
Glucagon-like peptide-1 receptor agonist	NA	NA	1806 (3)	909 (4)
Sodium-glucose cotransporter-2 inhibitors	NA	NA	96 (0.2)	47 (0.2)
Dipeptidyl peptidase-4 inhibitor	NA	NA	9156 (15)	2395 (12)
Insulin	NA	NA	NA	20 531 (100)
Other	NA	NA	428 (1)	283 (1)
Comorbidities				
Hypertension	233 116 (70)	44 088 (88)	53 866 (90)	19 157 (93)
Hyperlipidemia	209 826 (63)	41 425 (83)	49 901 (84)	17 489 (85)
Coronary heart disease	15 403 (5)	4357 (9)	4354 (7)	2478 (12)
Peripheral vascular disease	23 070 (7)	6917 (14)	6657 (11)	3651 (18)
Obesity	27 242 (8)	8221 (17)	10 716 (18)	5508 (27)
History of stroke or transient ischemic attack	26 547 (8)	6369 (13)	6273 (11)	3003 (15)
Chronic kidney disease	21 307 (6)	7179 (14)	7591 (13)	5891 (29)
Atrial fibrillation	34 792 (10)	7407 (15)	6904 (12)	3192 (16)
Congestive heart failure	18 422 (6)	6290 (13)	5672 (10)	4031 (20)

Abbreviations: IQR; interquartile range; NA, not applicable.

^a^ Baseline data were collected in the 270 days prior to the 90-day outcome period.

## Discussion

In this large Medicare cohort undergoing TJR, preoperative HbA_1c_ testing was performed in 26% to 43% of patients with diabetes and in only 5% of those without diabetes. Prior research has shown that an elevated HbA_1c_ level is associated with postoperative complications and, furthermore, that screening and addressing risk factors such as HbA_1c_ preoperatively may reduce complications, highlighting the importance of HbA_1c_ screening.^4,5,6^

Limitations of our study include possible misclassification of diabetes, as we relied on *ICD-9* codes, although we also used medication dispensing data to maximize accuracy. We were unable to assess for screening with fingerstick blood glucose or inpatient testing. Data were available from 2010 to 2014 and may not reflect current practice.

In a real-world clinical setting, hyperglycemia is often not screened for prior to TJR. Further study on the utility of perioperative hyperglycemia monitoring and optimization is warranted.
